# Metavirome Analysis and Identification of Midge-Borne Viruses from Yunnan Province, China, in 2021

**DOI:** 10.3390/v15091817

**Published:** 2023-08-26

**Authors:** Chenghui Li, Wei Wang, Xuancheng Zhang, Pengpeng Xiao, Zhuoxin Li, Peng Wang, Ning Shi, Hongning Zhou, Huijun Lu, Xu Gao, He Zhang, Ningyi Jin

**Affiliations:** 1 College of Agriculture, Yanbian University, Yanji 133002, China; lichenghuivip0056@163.com (C.L.); gaoxu@ybu.edu.cn (X.G.); 2 Changchun Veterinary Research Institute, Chinese Academy of Agricultural Sciences, Changchun 130122, China; 18104333623@163.com (X.Z.); 13043393036@163.com (Z.L.); pengwang202112@163.com (P.W.); huijun_lu@126.com (H.L.); 3 Institute of Virology, Wenzhou University, Wenzhou 325035, China; weiwang_wzu@126.com (W.W.); xiaopengpeng1010@126.com (P.X.); 4 College of Veterinary Medicine, Jilin University, Changchun 130062, China; shining17@jlu.edu.cn; 5 Yunnan Institute of Parasitic Diseases, Puer 665000, China; zhouhn66@163.com

**Keywords:** midge-borne viruses, metavirome analyses, phylogenetic analyses, virus isolation, virus identification

## Abstract

Midges are widely distributed globally and can transmit various human and animal diseases through blood-sucking. As part of this study, 259,300 midges were collected from four districts in Yunnan province, China, to detect the viral richness and diversity using metavirome analysis techniques. As many as 26 virus families were detected, and the partial sequences of bluetongue virus (BTV), dengue virus (DENV), and Getah virus (GETV) were identified by phylogenetic analysis and PCR amplification. Two BTV gene fragments, 866 bps for the VP2 gene of BTV type 16 and 655 bps for the VP5 gene of BTV type 21, were amplified. The nucleotide sequence identities of the two amplified BTV fragments were 94.46% and 98.81%, respectively, with two classical BTV-16 (GenBank: JN671907) and BTV-21 strains (GenBank: MK250961) isolated in Yunnan province. Furthermore, the BTV-16 DH2021 strain was successfully isolated in C6/36 cells, and the peak value of the copy number reached 3.13 × 10^7^ copies/μL after five consecutive BHK-21 cell passages. Moreover, two 2054 bps fragments including the E gene of DENV genotype Asia II were amplified and shared the highest identity with the DENV strain isolated in New Guinea in 1944. A length of 656 bps GETV gene sequence encoded the partial capsid protein, and it shared the highest identity of 99.68% with the GETV isolated from Shandong province, China, in 2017. Overall, this study emphasizes the importance of implementing prevention and control strategies for viral diseases transmitted by midges in China.

## 1. Introduction

Arthropod-borne viruses pose a serious threat to global public health. These viruses are mainly transmitted by mosquitoes, ticks, biting midges, and sandflies [1]. Midges are small blood-sucking insects, which are distributed all over the world and comprise a wide variety of species. Midges can spread a variety of diseases in the process of biting people and animals, including Akabane disease, Oropouche fever, Schmallenberg disease, epizootic hemorrhagic disease of deer, bovine ephemeral fever, epidemic hemorrhagic fever, bluetongue disease, catarrhal fever, and other diseases, which may seriously affect the health of people and animals [2,3,4]. To date, more than 50 viruses have been isolated from midges [5].

Traditional detection technology, such as reverse transcription polymerase chain reaction (RT-PCR) and real-time fluorescence quantitative PCR, mainly targets known viruses, so it is difficult to accurately identify unknown new viruses [6,7,8]. In contrast, Illumina sequencing technology has the advantage of detecting DNA sequences quickly and efficiently and performing large-scale reads. In particular, compared to third-generation sequencing nanopore technology, Illumina sequencing technology can achieve molecular barcodes of hundreds of complex samples each time with an accuracy of up to 99.9%. It has great application prospects in molecular genetic research, genomics, pathogen discovery, and identification [9,10].

Yunnan province, China, has a subtropical climate, which is very suitable for the survival and reproduction of various blood-sucking arthropods and even provides a good living environment for the preservation and transmission of most arboviruses. For example, more than 19 viral taxonomic families were identified from mosquitoes collected in different animal farms located in Yunnan province by using metagenomic sequencing techniques [11]. Moreover, multiple dengue virus (DENV) outbreaks occurred in Yunnan province during 2013–2021 [12]. On the other hand, Yunnan province borders many Southeast Asian countries and is an important distribution and activity area of midges, with a high risk of the cross-border transmission of various viruses [13]. Identifying the species of viruses carried and transmitted by midges in Yunnan province will provide additional information about viruses present in Southeast Asia. In this study, a large number of midge samples were collected from pig pens and cattle pens located in rural areas of four cities in Yunnan province, and the distribution of midge-borne viruses was monitored using metavirome analysis.

## 2. Results

### 2.1. Midge Sample Collection and Classification

Blood-sucking midges were the main targets of classification and identification of the samples, and a total of 9424 blood-sucking midges belonging to three genera and thirty-one species were identified (*Leptoconops*, *Culicoides*, and *Lasiohelea*) (Table S1). Four species of *Culicoides*, *C. jacobsoni*, *C. parahumeralis*, *C. palifer*, and *C. insignipennis*, were the dominant species in cattle and pig farms with a relatively abundant quantity. Moreover, it was found that the proportion of blood-sucking midges in the four sampling areas was 45.20% (2260/5000) in Chuxiong, 42.80% (2140/5000) in Lincang, 57.84% (2892/5000) in Dehong, and 42.64% (2132/5000) in Xishuangbanna. Among them, the proportion of Culicoides biting midges was 45.26% in Chuxiong, 45.92% in Lincang, 51.38% in Dehong, and 43.30% in Xishuangbanna.

### 2.2. Metaviral Sequencing and the Virome of Midges

To obtain accurate data for the midges, host sequences and barcode DNA were removed. A total of 12,739,524 reads and full-length 25,755,166 bps were obtained from the Illumina sequencing, and 66,613 contigs were assembled (Table S2). The assembled sequence reads accounted for 91.12% of the total reads, and the average length of the contigs was 239.81 NT, with 1.69% of the sequences annotated as viruses (1122/66,613), leaving 98.31% as unknown sequences (65,491/66,613). A total of 26 virus families were detected, and the clustering heat maps and statistical tables for the classification of the virus samples at the family level are shown in Figure S1 and Table 1. The sequencing results showed that the number of virus families (the content of virus sequences) in samples collected in Dehong, Chuxiong, Lincang, and Xishuangbanna was 23 (24.89%), 25 (15.23%), 22 (10.24%), and 22 (20.67%), respectively.

A total of 66,613 viral contigs were assembled using METSPADES software, including 6 DENV (Flaviviridae)-like contigs and 5 BTV (bluetongue virus, Reovirus)-like contigs. The DENV-like and BTV-like contigs shared 82.31–96.84% and 85.42–98.53% nucleotide identity with known DENV and BTV sequences, respectively.

### 2.3. PCR Validation of BTV Sequences

**Figure 1:**
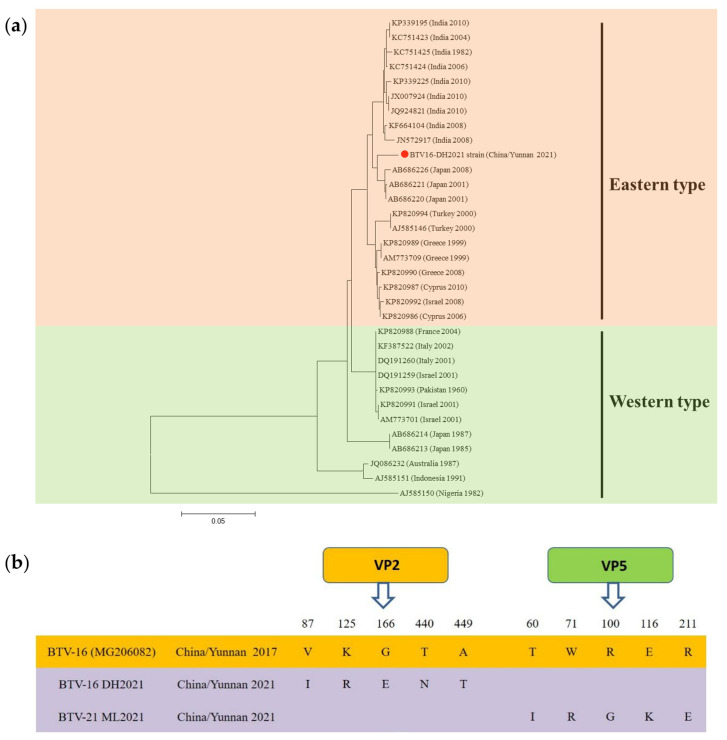
Phylogenetic tree based on the bluetongue virus type 16 (BTV-16) VP2 gene. (**a**) The application of the maximum likelihood method was used in Mega 7.0, where the bootstrap value was set to 1000. The solid red circle was used to mark the viral gene amplified in this study. (**b**) The VP2/VP5 amino acid sequences of BTV-16 DH2021/BTV-21 ML2021 were compared with the BTV-16 China/Yunnan 2017 strain.

Based on the results of viral contig alignment, two BTV gene fragments, 866 bps for the VP2 gene of BTV-16 and 655 bps for the VP5 gene of BTV-21, were amplified from samples collected in Chuxiong and Lincang using specific primers, respectively. Viral sequence analysis using BLASTN indicated that the amplified BTV-16 VP2 fragments shared the highest identity with BTV-16 isolated from Japan in 2008 (GenBank: AB686226), with 97.52% nucleotide identity. Compared with the classical BTV-16 strain isolated in Yunnan province (GenBank: JN671907), the nucleotide identity of amplified BTV-16 VP2 fragments was 94.46%. Moreover, compared with the classical BTV-21 strain isolated in Yunnan province (GenBank: MK250961), the nucleotide identity of amplified BTV-21 VP5 fragments was 98.81%. Furthermore, the nucleotide identity between the amplified BTV-21 VP5 fragments and the VP5 gene of the classical BTV-16 strain was 98.84%. Specifically, five mutations in the VP2 protein (87 aa, 125 aa, 166 aa, 440 aa, and 449 aa) and five mutations in the VP5 protein (60 aa, 71 aa, 100 aa, 116 aa, and 211 aa) were identified (Figure 1).

### 2.4. PCR Validation of DENV and GETV (Getah Virus) Sequences

**Figure 2:**
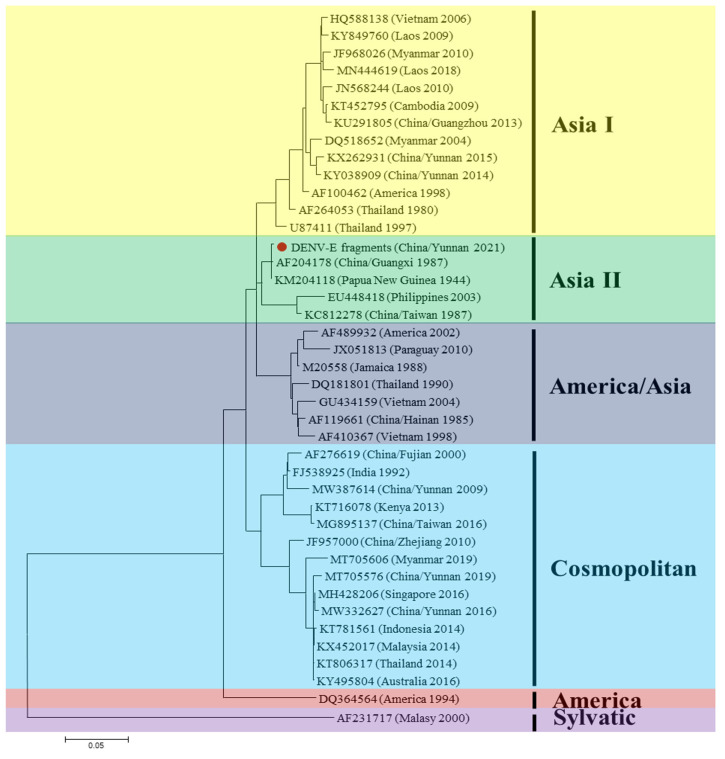
Phylogenetic tree based on the dengue virus (DENV) E gene. The application of the maximum likelihood method was used in Mega 7.0, where the bootstrap value was set to 1000. The solid red circle was used to mark the partial viral gene segments amplified in this study.

To further validate the results of the metavirome sequencing, DENV- and GETV-specific primers were designed and synthesized to amplify the viral-like sequences. Two 2054 bps nucleotide fragments including the E gene of DENV type II were obtained, and the nucleotide sequence identity between them was 99.62%. The phylogenetic analysis showed that the newly identified DENV sequences belong to genotype Asia II, and they shared the highest identity (99.51%) with the DENV strain isolated in New Guinea in 1944 (GenBank: KM204118) (Figure 2). 

**Figure 3:**
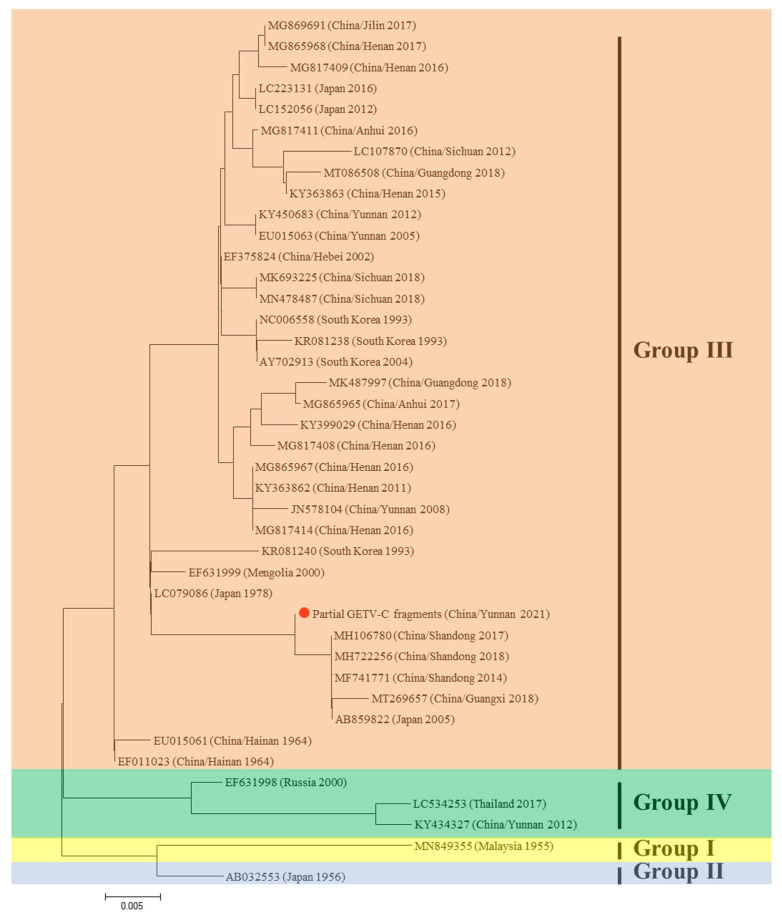
Phylogenetic tree based on the Getah virus (GETV) capsid protein gene. The application of the maximum likelihood method was used in Mega 7.0, where the bootstrap value was set to 1000. The solid red circle was used to mark the partial viral gene segments amplified in this study.

We did not identify GETV sequences in our metagenomic data. However, a partial sequence (656 bps) of the gene encoding the capsid protein of the GETV was amplified from the midge samples using nested PCR. Phylogenetic analysis indicated the newly derived GETV belonging to genotype III. BLASTN viral sequence analysis indicated that the amplified GETV fragment shared the highest identity with the GETV isolated from Shandong province, China, in 2017 (GenBank: MH106780), with a nucleotide sequence identity of 99.68% (Figure 3).

### 2.5. Isolation and Identification of the BTV-16 DH2021 Strain

**Figure 4:**
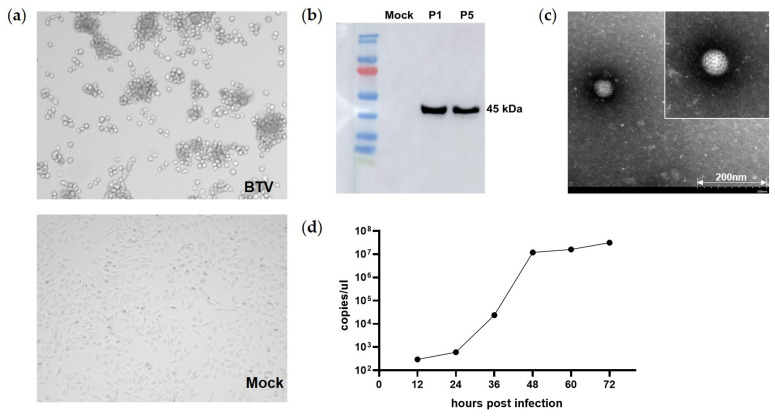
Isolation and identification of the BTV-16 DH2021 strain. (**a**) An obvious cytopathic effect (CPE) could be observed on the C6/36 cells (100×). (**b**) Identification of the effective expression of the BTV target protein using Western blot analysis. (**c**) BTV-16 DH2021 strain viral particles with a diameter of approximately 70 nm were visualized using negative stain electron microscopy. The scale of the original image is 200 nm. An enlarged view of a BTV particle is shown in the upper right corner. (**d**) The virus growth curve of BTV-16 DH2021 strain was determined within 72 h.

The sensitive cell lines were inoculated with the identified midge samples for virus isolation, but only BTV-16 DH2021 strain was successfully isolated. In particular, obvious CPE characteristics could be observed on C6/36 cells on the fourth day, such as obvious shrinkage, disintegration, and shedding of monolayer cells. The Western blot results showed that a band of the expected size appeared at 45 kDa. Then, the isolated virus was passaged on susceptible cells five consecutive times, and the target protein was effectively expressed with good stability. The growth curve of the fifth passage of BTV-16 DH2021 strain indicates active virus replication, as confirmed by a steady increase in genome copy number, with it reaching 3.13 × 10^7^ copies/μL at 72 h post inoculation. What is more, electron microscopy revealed that the virus particles were spherical with a diameter of approximately 70 nm, and there were numerous fibrillar protrusions on the surface of the virus (Figure 4).

## 3. Discussion

Midges are important insects with a worldwide distribution ranging from the Americas to Europe, Asia, and Oceania, with the exception of Antarctica and New Zealand [14]. Midges can transmit a wide range of pathogens such as bacteria, viruses, and parasites [15,16]. There are at least 5360 species of midges known in the world, including 1224–1530 species of Culicoides, 133 species of Leptomidges, 124 species of the subgenus Lasiocera of Forcipomyia, and 1 species of Australian midges [17]. In this study, a total of 259,300 midges were collected in Yunnan province, China. Blood-sucking midges were the main targets of classification and identification of the samples; however, we did not subdivide the species and genera of non-blood-sucking midges. According to the results of the sampling survey, three genera and thirty-one species were identified (*Leptoconops*, *Culicoides*, and *Lasiohelea*). In particular, four species of *Culicoides*, *C. jacobsoni*, *C. parahumeralis*, *C. palifer*, and *C. insignipennis*, were the dominant species on cattle and pig farms. Viral metagenomics has the potential advantage of discovering novel viral pathogens or recombinant types in various samples from animals or humans [18]. In addition, metagenomic sequencing is important for the discovery and identification of novel viruses [19,20]. In recent years, a variety of insect zoonotic viruses have been found in mosquitoes collected from Yunnan province using metagenomic analysis, including BTV, Banna virus (BAV), and Japanese encephalitis virus (JEV) [11]. Significantly, the natural tropical and subtropical climates also provide favorable conditions for midges’ propagation and virus transmission [21], which highlights the necessity of monitoring the virus diversity of midges using viral metagenomic analysis. In this study, metagenomic techniques were used to provide an initial exploration of viruses in midges in Yunnan province, and as many as 26 virus families were detected. Among these families, vertebrate viruses known to naturally infect mammals and replicate in mammalian cell lines included *Adenoviridae*, *Flaviviridae*, *Herpesviridae*, *Papillomaviridae*, *Reoviridae*, *Retroviridae,* and *Rhabdoviridae*. In 2018, metagenomics sequencing was carried out, and 41 virus families were detected in midges in Yunnan province, China [22]. Among them, there are 15 virus families that are consistent with the results detected in this study (*Tymoviridae*, *Poxviridae*, *Herpesviridae*, *Dicistroviridae*, *Flaviviridae*, *Peribunyaviridae*, *Baculoviridae*, *Rhabdoviridae*, *Parvoviridae*, *Hantaviridae*, *Nodaviridae*, *Hepadnaviridae*, *Partitiviridae*, *Reoviridae*, and *Ifaviridae*). The other 26 virus families were not detected in this study (*Orthomyxoviridae*, *Polydnaviride*, *Mimiviridae*, *Phycodnaviride*, *Retroviridae*, *Phenoviridae*, *Herpesviridae*, *Podoviride*, *Geminiviridae*, *Virgaviridae*, *Paramyxoviridae*, *Nairoviridae*, *Lipothrixviridae*, *Solinviridae*, *Ferraridae*, *Leviviridae*, *Carmotetraviridae*, *Caliviridae*, *Artiviridae*, *Secoviridae*, *Monaviridae*, *Gammsflixiviridae*, *Togaviride*, *Microviridae*, *Quadriviridae*, and *Picobirnaviridae*). In addition, there are 11 virus families (*Inoviridae*, *Luteoviridae*, *Adenoviridae*, *Siphoviridae*, *Myoviridae*, *Totiviridae*, *Autographiviridae*, *Circoviridae*, *Astroviridae*, *Tospoviridae*, and *Polycipiviridae*) that were not detected in 2018. Moreover, the sequences of plant viruses were identified, such as luteovirus, tospovirus and tymovirus, which may be due to the fact that midges feed on plant juice. Notably, metagenomics has also become a recognized and important tool for identifying and analyzing midge samples. In 2016, metagenomic studies on Senegal’s midges revealed the discovery of 11 virus families [23], and a total of 43 virus families were detected in a sample of Scottish midges in 2019 [24].

The BTV is one of the most important animal viruses that is transmitted by midges and mainly affects sheep. It can lead to reduced productivity and high mortality in ruminants, causing significant economic losses and indirectly reducing economic gains from the export trade of livestock products [25,26,27]. It was first detected and isolated within China in May 1979 in Shizong County of Yunnan province [28,29]. In recent years, there has been no large-scale epidemic outbreak of bluetongue in China. However, relevant studies have shown that a variety of serotypes of BTV are prevalent in Yunnan province, especially serotypes 1, 16, and 21, and foreign regional strains have invaded and undergone genetic recombination with the prevalent strains in China in the process of transmission [30]. The BTV-16 strain isolated in this study belonged to the Oriental topotype and showed the highest nucleotide sequence identity (97.52%) with the Japanese strain. Through comparison with the main epidemic strains of BTV-16 in China, it was found that there were five amino acid mutations in the VP2 protein. Whether the relevant mutations will cause changes in the biological characteristics of the virus remains to be further studied.

The GETV is a zoonotic virus transmitted by mosquitoes through bites, and it belongs to the *Togaviridae* family and *Alphavirus* genus, with the full-length genome of the virus being 11,000 to 12,000 nucleotides. GETV-infected animals, such as horses, pigs, and other livestock animals, can act as reservoirs for uninfected mosquitoes to acquire the virus. Multiple DENV outbreaks have occurred in Yunnan province, and mosquitoes have also been confirmed to be vectors of the DENV [31]. Here, the complete segment of the E gene of DENV II was successfully amplified for the first time from midges. However, we cannot exclude that the DENV identified from midge samples may have been present in a recent blood meal ingested by the midges or a very small number of mosquitoes may have been trapped in some of the samples. Therefore, it is unlikely that the midges may act as a novel vector. Interestingly, the gene fragments of the Newcastle disease virus were detected in samples from the Dehong district of Yunnan province in 2020 using viral metagenomics, which may suggest that the virus storage pool of midges is constantly expanding and needs to be taken seriously.

The partial capsid protein gene fragments of the GETV were analyzed through epidemiological surveillance, but they did not appear in metagenomics data. DENV and GETV were simultaneously detected, suggesting that these viruses may co-circulate in Yunnan province. Vero, C6/36, BHK-21 cells, and suckling mice were used for the isolation of GETV and DENV. Unfortunately, GETV and DENV were not successfully isolated from the midge samples. This may be due to an insufficient sample size or low viral load.

In this study, midge samples were collected from the Chuxiong, Xishuangbanna, Lincang, and Dehong districts of Yunnan province. The results suggest that there may be simultaneous transmission of multiple viruses in the midges in Yunnan province, and the sampling area should be further expanded to assess the diversity of midges. This study is helpful for identifying circulating viruses with zoonotic potential by using metagenomics technology.

## 4. Materials and Methods

### 4.1. Collection and Preparation of Midge Samples

**Figure 5:**
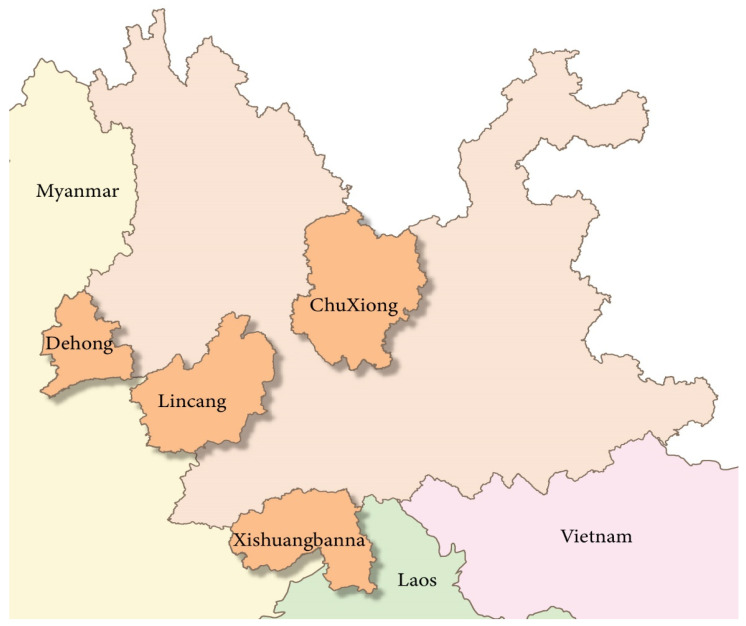
Regional distribution of midge samples collected in Yunnan province, China (orange-red). In total, 61,000 midges were collected in Dehong (coordinates: 24°43′22″ N 97°34′16″ E A: 261.0 m), 75,000 midges (coordinates: 25°24′26″ N 101°14′30″ E A: 1999.8 m) were collected in Chuxiong, 65,000 midges were collected in Xishuangbanna (coordinates: 21°26′17″ N 101°19′18″ E A: 575.0 m), and 58,300 midges were collected in Lincang (coordinates: 23°29′6″ N 98°50′5″ E A: 472.0 m).

According to the midge activity area, light traps were placed in pig pens and cattle pens to collect midges. A total of 259,300 midges were collected in rural areas of four cities in Yunnan province, China, in July, August, October and November 2021 (Figure 5). In order to understand the prevalence of midges in Yunnan province as a whole, the midge samples were mixed, treated, and analyzed uniformly. The midge samples were grouped according to the four collection areas and stored at −80 °C. Specifically, every 2000 to 3000 mixed midges were placed in the same cryopreservation tube. The number of mixed samples in each region was 44 in Dehong, 56 in Chuxiong, 49 in Xishuangbanna, and 40 in Lincang, totaling 189. Moreover, a sampling survey was also conducted to determine the proportion of blood-sucking and non-blood-sucking midges by randomly taking 5000 midges in each area and observing the morphology of the samples under a biological microscope. This study was approved by the Changchun Veterinary Research Institute.

Each midge sample was mixed with SM buffer (MeilunBio, Dalian, China) and then placed in a grinder for grinding. To remove midge debris and other substances, the samples were centrifuged at 13,000× *g* for 20 min, and the supernatant was used to extract viral nucleic acids. The extraction and reverse transcription of viral nucleic acids were performed as previously described [12]. Specifically, to remove the free nucleic acid and the contaminating host genomic DNA, 14 U Turbo DNase (Ambion, Austin, TX, USA), 25 U Benzonase Nuclease (Novagen, San Diego, CA, USA), 20 U RNase I (Fermentas, Ontario, Canada), and 10 × DNase buffer (Ambion) were added to 127 μL of the supernatants to a final volume of 150 μL, followed by digestion at 37 °C for 1 h. The viral nucleic acid was extracted using a virus nucleic acid kit (Bioer Technology, Hangzhou, China) according to the manufacturer’s instructions. The total viral nucleic acids were reverse transcribed using anchored random primers (Table S3) and Superscript III reverse transcriptase (Invitrogen, Carlsbad, CA, USA).

### 4.2. Metagenomic Sample Preparation and Metaviral Sequencing

The methods used for metagenomic sample preparation and metaviral sequencing were in reference to previous research [12]. Specifically, the samples were divided into four groups according to the collection site, each group was integrated into one metavirome sample, and barcode primers 1–4 were added. Each metavirome sample was run with the same Illumina. The details of the barcode DNA used in the metagenomic analysis are shown in Table S3.

### 4.3. Primer Designed for Specific Viruses

Sequence information for NR (Non-Redundant Protein Sequence Database) library alignments and virus classification details for ICTV (International Committee on the Taxonomy of Viruses) reports were downloaded from GenBank (https://www.ncbi.nlm.nih.gov/genbank/, accessed on 1 July 2023) and then aligned using Mega 7 software. The retention region (preferably 400–800 bps in length) was truncated, and the region was subjected to primer design with Primer Premier 5 software, taking precedence over nested primers and primer sequence information provided in Table S4. Among them, four pairs of primers were designed for BTV, two pairs of primers were designed for GETV, and one pair of primers was designed for DENV.

### 4.4. Virus Isolation and Identification

The BHK-21 (5% fetal bovine serum in Dulbecco’s modified Eagle’s medium, HyClone, Logan, UT, USA) and C6/36 (10% fetal bovine serum in modified Eagle’s medium, HyClone, USA) monolayer cells were supplemented with 50 μL of the supernatant of midge tissue grinding liquid and incubated at 37 °C or 28 °C for 6 days, respectively.

The cytopathic effect (CPE) was observed every day, and more than three blind passages of cell culture were performed. The isolated bluetongue virus (BTV DH2021 strain) was detected by PCR using specific primers (Table S4). The BTV-VP7 monoclonal antibody (Laboratory preservation, Changchun, China) was used as the primary antibody, and an HRP-conjugated goat anti-mouse antibody (Transgene, Beijing, China) was used as the secondary antibody for the Western blot analysis. In addition, the BTV particles were visualized using negative stain electron microscopy, which was prepared using the supernatants of infected BHK-21 cells mixed with 6.1% paraformaldehyde mounted on copper grids and treated with 3% phosphotungstic acid. The virus titer growth curve of BTV in the BHK cells was detected according to the methods reported in the literature [32].

## 5. Conclusions

Among the 259,300 collected midges from four regions of Yunnan province in 2021, four species of *Culicoides* (*C. jacobsoni*, *C. parahumeralis*, *C. palifer*, and *C. insignipennis*), were the dominant species on cattle and pig farms. In addition, 26 virus families were detected by metavirome analysis, and partial sequences of BTV, DENV, and GETV were identified. Furthermore, a BTV-16 DH2021 strain was successfully isolated. It is necessary to pay close attention to the global trend of midge-borne diseases and the diversity of midge viral populations.

## Figures and Tables

**Table 1:** Number of viral families found using metavirome analysis at four localities.

Family	Chuxiong	Dehong	Lincang	Xishuangbanna
Adenoviridae	12	1	1	1
Astroviridae	1	1	4	1
Autographiviridae	0	1	2	2
Baculoviridae	23	11	23	230
Circoviridae	4	9	4	3
Dicistroviridae	219	30	35	31
Flaviviridae	6	1	1	1
Hantaviridae	0	1	0	0
Hepadnaviridae	1	1	1	1
Herpesviridae	26	5	5	3
Iflaviridae	7	4	16	4
Inoviridae	1	1	0	0
Luteoviridae	1	1	0	1
Myoviridae	8	2	3	4
Nodaviridae	0	1	0	0
Partitiviridae	2	2	2	2
Parvoviridae	1	1	1	2
Peribunyaviridae	2	2	1	9
Polycipiviridae	33	26	55	24
Poxviridae	5	3	4	2
Reoviridae	1	1	2	1
Rhabdoviridae	19	58	34	15
Siphoviridae	7	3	4	5
Tospoviridae	1	0	2	0
Totiviridae	11	5	6	7
Tymoviridae	11	8	6	9

## Data Availability

The amplified gene sequences have been listed in the supplementary file.

## Supplementary Materials

The following supporting information can be downloaded at: https://www.mdpi.com/article/10.3390/v15091817/s1, Table S1: Details of the composition of the midge samples analyzed by morphological observation; Table S2: The results of metagenomic analysis and Illumina sequencing of midges; Table S3: Details of barcode DNA used in metagenomic analysis; Table S4: Details of primer pairs used for identification by PCR; Figure S1: Clustered heat map of the classification of the midge samples at the family level. Section S1: The amplified gene sequences.
