# Supplementary material for: Metavirome Analysis and Identification of Midge-Borne Viruses from Yunnan Province, China, in 2021
Source: Viruses. 2023 Aug 26;15(9):1817. doi: 10.3390/v15091817 (PMC10535587; doi:10.3390/v15091817)
Supplement: Supplementary file 1 [file viruses-15-01817-s001.zip › viruses-2513906-supplementary.pdf]

Table S1. Details of the composition of the midge samples analyzed by morphological observation.

Species and genera of midges	Dehong	Chuxiong	Lincang	Xishuangbanna	Total	
					Number	Percentage
Le.ascia		+		+	8	0.08%
Le. menglaensis			+	+	112	1.18%
Le. yunhsienensis			+	+	239	2.53%
Le. yunnanensis	+	+	+	+	659	6.99%
La. abdita		+	+	+	23	0.24%
La. taiwana	+		+		7	0.07%
La. phototropia	+			+	11	0.01%
La. uncusipenis			+	+	37	0.39%
La. tenuidentis	+	+			52	0.55%
La. nemorosa			+		3	0.03%
La. multidentis			+		238	2.50%
C. arakawai	+				13	0.13%
C. yunnanensis	+	+	+	+	302	3.18%
C. toshiokai	+		+		21	0.22%
C. menglaensis				+	204	2.15%
C. elbeli		+		+	39	0.41%
C. gentiloides		+	+	+	126	1.33%
C. holcus		+	+		105	1.11%
C. homotomus		+		+	19	0.21%
C. manhauensis	+		+		76	0.81%
C. malayae		+	+	+	29	0.30%
C. parabarnetti		+	+		621	6.58%
C. kinabaluensis	+			+	891	9.45%
C. lansangensis		+	+		794	8.42%
C. jacobsoni	+		+		1,227	13.01%
C. parahumeralis	+	+		+	1,027	10.89%
C. palpifer	+		+		992	10.52%
C. peregrinus	+			+	241	2.55%
C. insignipennis		+		+	1,209	12.83%
C. pastus			+		42	0.44%
C. malayae	+				57	0.62%
Total	2,892	2,260	2,140	2,132	9,424	100%

*Le: *Leptoconops*; C: *Culicoides*; La: *Lasiohelea*; “+” : epidemic areas.

Table S2. The results of metagenomic analysis and Illumina sequencing of midges.

Name	Species	Number	Location	Total length	Contig Number	N50 Length	Max Contig Length
Sample I	Midge Community	61,000	Dehong	4,955,444	11,227	477	4,961
Sample II	Midge Community	75,000	Chuxiong	7,503,337	20,996	341	5,343
Sample III	Midge Community	65,000	Xishuangbanna	7,365,464	19,797	357	3,665
Sample IV	Midge Community	58,300	Lincang	5,930,921	14,593	402	5,969
Total/Average	-	259,300	-	25,755,166	66,613		

Table S3. Details of barcode DNA used in metagenomic analysis.

Primer Type	Primers Number	Sequence (5'-3')
Anchored Random Primers	RT1	GCCGGAGCTCTGCAGATATCNNNNNN
	RT2	GTATCGCTGGACACTGGACCNNNNNN
	RT3	ATCGTCGTCGTAGGCTGCTCNNNNNN
	RT4	CGTAGATAAGCGTCCGCTCNNNNNN
Barcode Primers	Primer1	GCCGGAGCTCTGCAGATATC
	Primer2	GTATCGCTGGACACTGGACC
	Primer3	ATCGCGTCGTAGCTGCTC
	Primer4	CGTAGATAAGCGGTCGGCTC

Table S4. Details of primer pairs used for identification by PCR.

Primer Name	Sequence (5'-3')	Products (bp)
BTV16E-F	GGGATGTTTTCAAACACGT	
BTV16E-R	GATGTGTGTTCGCTCGAAG	434
BTV21-VP5F	TACGATTGCGGAAGATGGG	
BTV21-VP5R	TCTTGAATCGCTTCCTCCTG	656
BTV16-VP1-1-F	TATGATGTTTCGCAAGTGGTAGT	
BTV16-VP1-1-R	AATGCCTCGTAAATGCCTGTGGG	865
BTV16-VP2-2-F	TATGATGTTTCGCAAGTGGTAGT	
BTV16-VP2-2-R	AATGCCTCGTAAATGCCTGTGGG	951
DENV-D2-E-F	GACCTTGGTGARTTGTGTAAG	
DENV-D2-E-R	CARTCTTGTTACTGAGCGGA	2054
GETV-F1	AGCACCGAAGAAGCCGAAGA	
GETV-R1	CGTTGGCTAAGACGCACATCA	656
GETV-F2	AGTCGGGGATAAAGTGATGAAGC	
GETV-R2	GTCCTTTGTTGTCGAAGATCGG	283

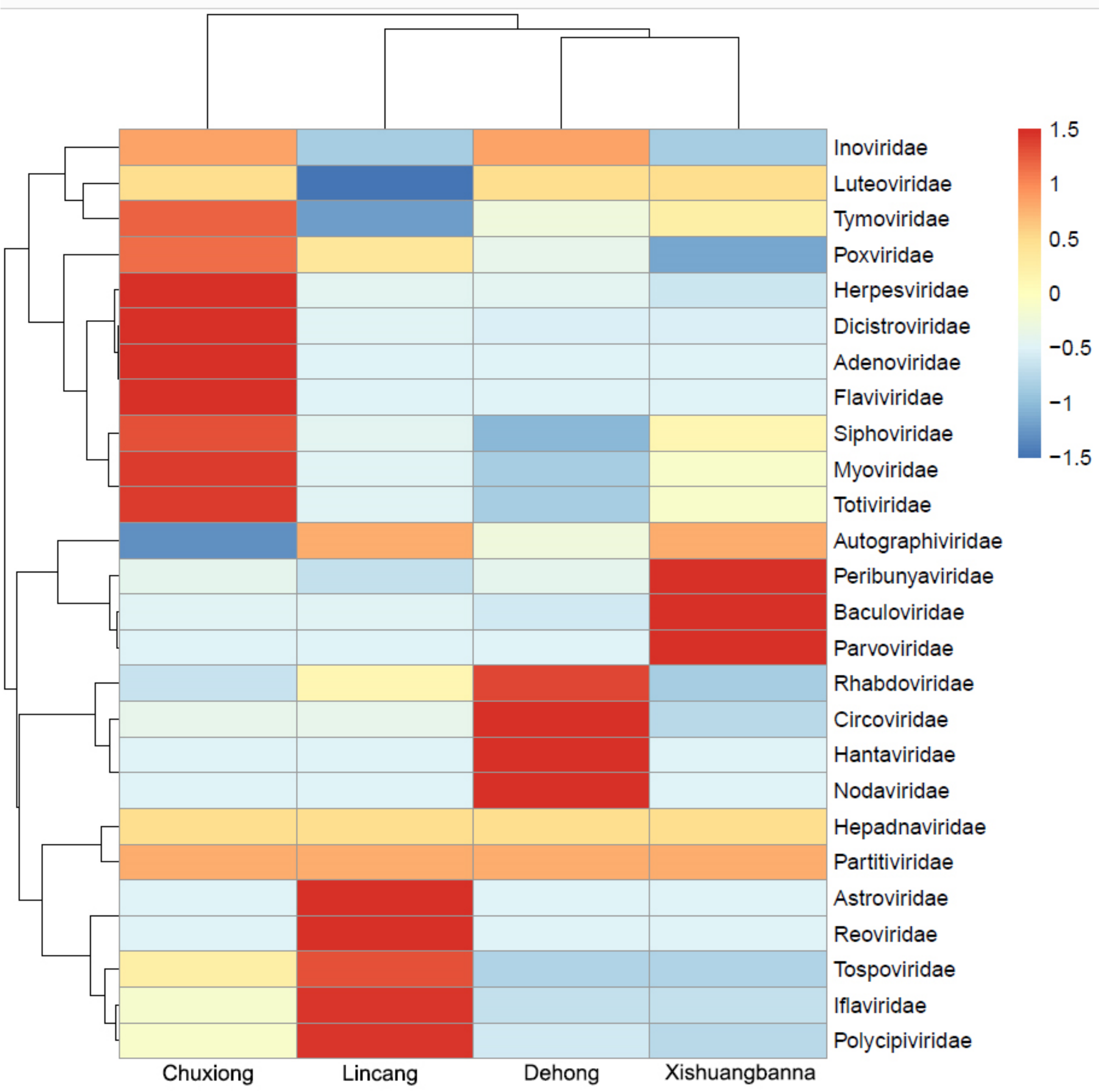

Figure S1. Clustered heat map of the classification of the midge samples at the family level. Viral sequences are classified according to virus family, with brighter colors indicating greater abundance.

Section S1. The amplified gene sequences.

BTV16-VP2(866 bps):

ATTCTAGATATGTCCATTGCGTGTTACGATATGAAAAAGAGTATGATGAAGA
AGGATGGGGTCGATTTTGTATCAAACACAAAATGGTTAGAATGGATGATAG
GAGATTCGATGGATGTGCAACCTCTAAGGGTCCAACTGAAAGAAGATCAC
AGTACAATACAATATGGGATGTTCTCAAACACGTTACACATCGATTCGAGGA
AAGCGGATACCACGTCATATCATACGATTGCTGTAGAATCAAAAGGGGAAC
GTGGGTGCTGTCACGTTCACACGGCAATTTGGAATCACATGGTACGTAATC
ACTTGTTTAACGCCGTCCAAGAGGCGTGCTACGTGTTTAAGCCGACGTATG
ATTTAATAGTGATTGGTGAAAAACAGAATCGTGAGGATGAATTTAGGATTG
GCGAACACAATTTTTATACCATAACACGGAATCACCACATGCGTTTAGGCG
ATAACGCGTATAATCAGTTCATGAAAGGTTTGGTTCAGTTGCGCGTGGCGG
GAGTGACACCGAATGTAATACGAGAGGAAATGGCCGCTTTAGACGCGATAA
GGGATACTTGGATAGGAGGGAACTTCGAGCGAACACACATTAAATCTCTTG
AAATATGTAAGTTATTATCCAGCATTGGAAGAAAGATGGTTAATATGGAGGA
GGAACCAAAGGATGAAAGAGACCTATCAGTTAAATTTCAATTTAAACTCGA
CGACAAATTTTCAACAACCGATCCGGAAAGAAACGTCATCTTTACACATAA
AACACACCGTACGAATCAAGATCGTTTCTATGTGTTCTAATGATTGCGGCGT
CGGACACAAATAACGGTAGAGTATGGTGGTCAAACCCTTATCCGTGT

BTV21-VP5(655 bps):

TACGATTGCGGAAGATGGGTAAAATCATCAAATCACTAAGCCGTTTCGGCA
AGAAGGTTGGGAACGCGTTAACGTCTAGTACAGCGAAGAAAATCTATAGC
ACCATAGGAAAGGCGGCGGAAAGGTTCGCGGAAAGTGAGATTGGGTCCGC
GGCAATTGATGGATTAGTTCAAGGTAGTGTTCACTCAATCATGACCGGGGA
GTCATATGGCGAATCGGTGAAACAAGCTGTACTGCTGAACGTTCTGGGGGC
GGGGGACGAGATTCCTGATCCGTTAAGCCCGGGGGAACGCGGCATCCAGA
TGAAGATCAAGGAGATTGAAGAAGAGCAAAGAAACGAATTGGTAAGGCTG
AAGCATGGGAAGGAGATTACAAAGAAATTTGGCGAAGAACTAGAGGAGAT
ATATCAATTTATGAATGGTGAGGTGAAGGATGAGGAAGAGCAGGAAGAGC
AATATAAAGTGCTATGTAAGGCGGTTAACTCGTACGAAAAATTACTTATAGC
TGAAAATGATAAGATGCACATATTAGCTCGCGCATTGCAGAGGGAGGCAGC
TGAAAGGACTGAAGCTGAATCAACAATGGTTAAAGAGTACAGGCAGAAAA
TTGATGCACTGAAGGCTGCCATCGAAATCGAACGGGATGGGATGCAGGA

DENV-E-1(2054 bps):

TACGATTCACTATAGGGCGAATTGAATTTAGCGGCCGCGAATTCGCCCTTGA
CCTTGGTGAATTGTGTGAAGATACAATCACGTACAAGTGTCCTCTTCTCAG
GCAGAATGAACCAGAAGACATAGATTGTTGGTGCAACTCTACGTCCACATG
GGTAACTTATGGGACGTGTACCACTACAGGAGAACACAGAAGAGAAAAAA
GATCAGTGGCACTCGTTCCACATGTGGGAATGGGACTGGAGACACGAACT
GAAACATGGATGTCATCAGAAGGGGCCTGGAAACATGCCCAGAGAATTGA
AACTTGGATCTTGAGACATCCAGGCTTTACCATGATGGCAGCAATCCTGGC
ATACATCATAGGAACGACACATTTCCAAAGAGCCCTGATTTTCATCTTACTG
ACAGCTGTCGCTCCTTCAATGACAATGCGTTGCATAGGAATATCAAATAGAG
ACTTTGTAGAAGGGGTTTCAGGAGGAAGCTGGGTTGACATAGTCTTAGAAC

ATGGAAGCTGTGTGACGACGATGGCAAAAAACAAACCAACATTGGATTTT
GAACTGATAAAAACAGAAGCCAAACAACCTGTCACTCTAAGGAAGTACTG
TATAGAGGCAAAGCTGACCAACACAACAACAGATTCTCGCTGCCCAACAC
AAGGAGAACCCAGCCTAAATGAAGAGCAGGACAAAAGGTTCGTCTGCAA
ACACTCCATGGTGGACAGAGGATGGGGAAATGGATGTGGATTATTTGGAAA
AGGAGGCATTGTGACCTGTGCTATGTTCACATGCAAAAAGAACATGAAAG
GAAAAGTCGTGCAACCAGAAAACTTGGAATACACCATTGTGATAACACCTC
ACTCAGGGGAAGAGCATGCAGTCGGAAATGACACAGGAAAACATGGCAA
GGAAATCAAAATAACACCACAGAGTTCCATCACAGAAGCAGAGTTGACAG
GCTATGGCACTGTCACGATGGAGTGCTCTCCGAGAACGGGCCTCGACTTCA
ATGAGATGGTGTTGCTGCAAATGGAAAATAAAACTGGGCTGGTGCACAGG
CAATGGTTCCTAGACCTGCCGTTGCCATGGCTGCCCGGAGCGGACACACAA
GGATCAAATTGGATACAGAAAGAGACATTGGTCACTTTCAAAAATCCCCAT
GCGAAGAAACAGGATGTTGTTGTTTTGGGATCCCAAGAAGGGGCCATGCA
CACAGCACTCACAGGGGCCACAGAAATCCAGATGTCATCAGGAAACTTAC
TGTTCACAGGACATCTCAAGTGCAGGCTGAGGATGGACAAACTACAGCTC
AAAGGAATGTCATACTCTATGTGCACAGGAAAGTTTAAAGTTGTGAAGGAA
ATAGCAGAAACACAACATGGAACAATAGTTATCAGAGTACAATATGAAGGG
GACGGTTCTCCATGTAAGATCCCTTTTGAGATAATGGATTTGGAAAAAAGA
CATGTTTTAGGTCGCCTGATTACAGTCAACCCAATCGTAACAGAAAAAGAT
AGCCCAGTCAACATAGAAGCAGAACCTCCATTCGGAGACAGCTACATCATC
ATAGGAGTAGAGCCGGGACAATTGAAGCTCAACTGGTTTAAGAAAGGAAG
TTCTATCGGCCAAATGTTTGAGACAACAATGAGGGGAGCGAAGAGAATGG
CCATTTTAGGTGACACAGCTTGGGATTTTGGATCCCTGGGAGGAGTGTTTA
CATCTATAGGAAAGGCTCTCCACCAAGTTTTCGGAGCAATCTATGGGGCTG
CCTTCAGTGGGGTCTCATGGACTATGAAAATCCTCATAGGAGTCATTATCAC
ATGGATAGGAATGAATTCACGCAGCACCTCACTGTCTGTGTCACTAGTATTG
GTGGGAGTCGTGACGCTGTATTTGGGAGTTATGGTGCAGGCCGATAGTGGT
TGCGTTGTGAGCTGGAAAAACAAAGAACTGAAGTGTGGCAGTGGGATTTT
CATCACAGACAACGTGCACACATGGACAGAACAATACAAGTTCCAACCAG
AATCCCCTTCAAAACTAGCTTCAGCTATC

DENV-E-2(2054 bps):

AATACGATTCACTATAGGGCGAATTGAATTTAGCGGCCGCGAATTCGCCCTT
GACCTTGGTGAATTGTGTGAAGATACAATCACGTACAAGTGTCCTCTTCTC
AGGCAGAATGAACCAGAAGACATAGATTGTTGGTGCAACTCTACGTCCAC
ATGGGTAACTTATGGGACGTGTACCACTACAGGAGAACACAGAAGAGAAA
AAAGATCAGTGGCACTCGTTCCACATGTGGGAATGGGACTGGAGACACGA
ACTGAAACATGGATGTCATCAGAAGGGGCCTGGAAACATGCCCAGAGAAT
TGAAACTTGGATCTTGAGACATCCAGGCTTTACCATGATGGCAGCAATCCT
GGCATACATCATAGGAACGACACATTTCCAAAGAGCCCTGATTTTCATCTTA
CTGACAGCTGTCGCTCCTTCAATGACAATGCGTTGCATAGGAATATCAAATA
GAGACTTTGTAGAAGGGGTTTCAGGAGGAAGCTGGGTTGACATAGTCTTA
GAACATGGAAGCTGTGTGACGACGATGGCAAAAAACAAACCAACATTGGA
TTTTGAACTGATAAAAACAGAAGCCAAACAACCTGTCACTCTAAGGAAGT

ACTGTATAGAGGCAAAGCTGACCAACACAACAACAGATTCTCGCTGCCCA
ACACAAGGAGAACCCAGCCTAAATGAAGAGCAGGACAAAAGGTTCGTCT
GCAAACACTCCATGGTGGACAGAGGATGGGGAAATGGATGTGGATTATTTG
GAAAAGGAGGCATTGTGACCTGTGCTATGTTCACATGCAAAAAGAACATG
AAAGGAAAAGTCGTGCAACCAGAAAACTTGGAATACACCATTGTGATAAC
ACCTCACTCAGGGGAAGAGCATGCAGTCGGAAATGACACAGGAAAACATG
GCAAGGAAATCAAAATAACACCACAGAGTTCCATCACAGAAGCAGAGTTG
ACAGGCTATGGCACTGTCACGATGGAGTGCTCTCCGAGAACGGGCCTCGA
CTTCAATGAGATGGTGTTGCTGCAAATGGAAAATAAAACTGGGCTGGTGCA
CAGGCAATGGTTCCTAGACCTGCCGTTGCCATGGCTGCCCGGAGCGGACAC
ACAAGGATCAAATTGGATACAGAAAGAGACATTGGTCACTTTCAAAAATCC
CCATGCGAAGAAACAGGATGTTGTTGTTTTGGGATCCCAAGAAGGGGCCAT
GCACACAGCACTCACAGGGGCCACAGAAATCCAGATGTCATCAGGAAACT
TACTGTTCACAGGACATCTCAAGTGCAGGCTGAGGATGGACAAACTACAG
CTCAAAGGAATGTCATACTCTATGTGCACAGGAAAGTTTAAAGTTGTGAAG
GAAATAGCAGAAACACAACATGGAACAATAGTTATCAGAGTACAATATGAA
GGGGACGGTTCTCCATGTAAGATCCCTTTTGAGATAATGGATTTGGAAAAA
AGACATGTTTTAGGTCGCCTGATTACAGTCAACCCAATCGTAACAGAAAAA
GATAGCCCAGTCAACATAGAAGCAGAACCTCCATTCGGAGACAGCTACATC
ATCATAGGAGTAGAGCCGGGACAATTGAAGCTCAACTGGTTTAAGAAAGG
AAGTTCTATCGGCCAAATGTTTGAGACAACAATGAGGGGAGCGAAGAGAA
TGGCCATTTTAGGTGACACAGCTTGGGATTTTGGATCCCTGGGAGGAGTGT
TTACATTTATAGGAAAGGCTCTCCACCAAGTTTTCGGAGCAATCTATGGGGC
TGCCTTCAGTGGGGTCTCATGGACTATGAAAATCCTCATAGGAGTCATTATC
ACATGGATAGGAATGAATTCACGCAGCACCTCACTGTCTGTGTCACTAGTAT
TGGTGGGAGTCGTGACGCTGTATTTGGGAGTTATGGTGCAGGCCGATAGTG
GTTGCGTTGTGAGCTGGAAAAACAAAGAACTGAAGTGTGGCAGTGGGATT
TTCATCACAGACAACGTGCACACATGGACAGAACAATACAAGTTCCAACC
AGAATCCCCTTCAAAACTAGCTTCAGCTA

GETV(656 bps):

CGAAGAAGAAGCCACAAAAAGCGAAGGCTAAGAAAAACGAACAGCAAA
AGAAAAACGAGAACAAGAAACCACCACCTAAGCAGAAGAATCCGGCTAA
GAAGAAGAAACCAGGAAAAAGGGAACGCATGTGCATGAAGATAGAGAAT
GATTGCATCTTCGAGGTCAAGCTTGACGGTAAGGTCACGGGCTACGCCTGC
CTAGTCGGGGATAAAGTGATGAAGCCGGCACACGTTAAAGGTGTGATCGA
CAACCCCGACCTAGCGAAGCTTACCTACAAGAAATCGAGCAAGTATGACCT
AGAGTGCGCCCAGATACCGGTGCACATGAAGTCAGATGCTTCAAAGTACA
CCCATGAAAAACCAGAAGGGCACTACAATTGGCATCACGGTGCAGTGCAG
TACAGCGGTGGCAGGTTCACAATCCCGACAGGCGCAGGTAAACCAGGAGA
CAGCGGCCGGCCGATCTTCGACAACAAAGGACGTGTGGTGGCCATTGTCC
TGGGAGGGGCCAACGAAGGAGCCAGGACTGCCCTATCTGTCGTGACCTGG
ACCAAAGACATGGTCACACGGTACACCCCAGAAGGAACAGAAGAATGGTC
CGCCGCCTTGATGATGTGCGTCTTAGCCAACGTTACATTCCCATGCTCAGAG
CCCGCA
